# Metagenomic next-generation sequencing for mixed pulmonary infection diagnosis

**DOI:** 10.1186/s12890-019-1022-4

**Published:** 2019-12-19

**Authors:** Jiahui Wang, Yelei Han, Jing Feng

**Affiliations:** Respiratory Department, Tianjin Medical University General Hospital, Tianjin, 300052 China

**Keywords:** mNGS, Diagnosis, Mixed pulmonary infection

## Abstract

**Background:**

Metagenomic next-generation sequencing (mNGS) is emerging as a promising technique for pathogens detection. However, reports on the application of mNGS in mixed pulmonary infection remain scarce.

**Methods:**

From July 2018 to March 2019, 55 cases were enrolled in this retrospective analysis. Cases were classified into mixed pulmonary infection (36 [65.5%]) and non-mixed pulmonary infection (19 [34.5%]) according to primary diagnoses. The performances of mNGS and conventional test on mixed pulmonary infection diagnosis and pathogen identification were compared.

**Results:**

The sensitivity of mNGS in mixed pulmonary infection diagnosis was much higher than that of conventional test (97.2% vs 13.9%; *P <* 0.01), but the specificity was the opposite (63.2% vs 94.7%; *P =* 0.07). The positive predictive value of mNGS was 83.3% (95% CI, 68.0–92.5%), and the negative predictive value was 92.3% (95% CI, 62.1–99.6%). A total of 5 (9.1%) cases were identified as mixed pulmonary infection by both conventional tests and mNGS, however, the pathogens identification results were consistent between these two methods in only 1 (1.8%) case. In summary, the pathogens detected by mNGS in 3 (5.5%) cases were consistent with those by conventional test, and only 1 (1.8%) case was mixed pulmonary infection. According to our data, mNGS had a broader spectrum for pathogen detection than conventional tests. In particular, application of mNGS improved the diagnosis of pulmonary fungal infections. Within the 55 cases, mNGS detected and identified fungi in 31 (56.4%) cases, of which only 10 (18.2%) cases were positive for the same fungi by conventional test. The most common pathogen detected by mNGS was *Human cytomegalovirus* in our study, which was identified in 19 (34.5%) cases of mixed pulmonary infection*. Human cytomegalovirus* and *Pneumocystis jirovecii,* which were detected in 7 (12.7%) cases, were the most common co-pathogens in the group of mixed pulmonary infection.

**Conclusions:**

mNGS is a promising technique to detect co-pathogens in mixed pulmonary infection, with potential benefits in speed and sensitivity.

**Trial registration:**

**(**retrospectively registered): ChiCTR1900023727. Registrated 9 JUNE 2019.

## Background

Pulmonary infection is a leading cause of death and morbidity worldwide [1]. The risk of mixed pulmonary infection is high, especially in immunocompromised patients such as patients with hematological malignancies. Mixed pulmonary infection was defined when two or more infectious pathogens were indentified. Compared to patients with monomicrobial pulmonary infection, patients with polymicrobial pulmonary infection may have different antibiotic spectrums and more severe clinical manifestation. Diagnosis of polymicrobial infection must be as accurate as possible, because combined treatment has many potential side effects [2]. However, fast and accurate infection diagnose is challenging due to the limitations of current conventional tests in terms of sensitivity, speed and spectrum for pathogen detection [3, 4].

Next-generation sequencing (NGS), also termed high-throughput or massively parallel sequencing, is a technology that allows for thousands to billions of DNA fragments to be simultaneously and independently sequenced. The applications of NGS in clinical microbiological testing are manifold, including metagenomic NGS (mNGS), which allows for an unbiased detection of pathogens [5]. When applied to clinical practice, Qing Miao et al. reported that the sensitivity and specificity of mNGS for diagnosing infectious disease were 50.7 and 85.7% respectively. mNGS outperformed culture-depend methods, especially for the detection of *Mycobacterium tuberculosis* (MTB), viruses, anaerobes and fungi. Furthermore, mNGS is less affected by prior antibiotic exposure [6]. In addition, mNGS could offer an improved detection of pulmonary infectious pathogens in lung biopsy tissues, with potential benefits in speed and sensitivity [7]. However, reports on the use of mNGS in mixed pulmonary infection remain scarce. Most studies on mNGS focused on the diagnosis of single infection.

In this study, we evaluated the performance of this approach in the diagnosis of mixed pulmonary infection. The mNGS results were compared with those from conventional laboratory-based diagnostic methods. Our results indicated that mNGS benefited the efficiency of co-pathogens detection.

## Materials and methods

### Specimen collection and processing

Pulmonary biopsy and bronchoalveolar lavage fluid (BALF) of patients with suspected pulmonary infection in Tianjin Medical University General Hospital were collected by bronchoscopy according to standard procedures. 3 to 5 lung biopsy specimens were taken from every patient to meet the needs of pathology and pathogen examination. Qualified BALF meets the following conditions: no airway secretions in BALF; recovery rate > 40%, surviving cells accounting for more than 95%; red blood cells < 10% (excluding trauma/bleeding factors), epithelial cells < 3 to 5% and intact smear cells without deformation. Specimens from a total of 55 cases collected between July 2018 and March 2019 were enrolled in this study. The lung biopsies were sent to histopathology laboratories within 2 h of collection. The histopathology laboratory used standard methods for processing clinical specimen. Periodic acid-Schiff (PAS) staining, acid-resistant staining and hexamine silver staining were carried out. A portion of bronchoalveolar lavage fluid was used for culture of aerobic bacteria, anaerobic bacteria, fungi, viruses and mycobacteria. Another part of the bronchoalveolar lavage fluid was used for Xpert MTB, galactomannan (GM) test and smear. Gram staining, KOH testing and Ziehl-Neelsen staining were used to identify bacteria, fungi and mycobacteria by smear microscopy. The remaining specimens were stored at − 80 °C for mNGS. An additional file shows diagnostic flow for mixed pulmonary infection [see Additional file 1: Figure S1]. Written informed consent was obtained from the patients.

### Metagenomic next-generation sequencing and analysis

DNA of samples were extracted from BALF and tissue homogenates with a TIANamp Micro DNA Kit (DP316, TIANGEN BIOTECH) according to the manufacturer’s recommendation. DNA libraries were constructed as previously described [6]. Low-quality and short (length < 35 bp) reads were removed for generating high-quality sequencing data. Burrows-Wheeler Aligner software was used for mapping to a human reference (hg19) to identify human sequence data. Microbial genome databases were used to classify the remaining data [6, 8, 9]. The classification reference databases were downloaded from NCBI (ftp://ftp.ncbi.nlm.nih.gov/genomes/). The infectious pathogen was determined if it met any of the following thresholds: (i) culture and/or histopathological examination positive of bacteria, virus or fungi, invasive pulmonary aspergillosis (IPA) was defined according to modified EORTC criteria using galactomannan antigen and PCR as well [10]; (ii) at least 50 unique reads from a single species of bacteria, virus or fungi; for pathongen with unique reads less than 50, it still can be diagnosed as infectious pathogen with the consistent clinical situation; (iii) at least one unique read from *Mycobacterium tuberculosis* complex (MTBC)*.* Mixed pulmonary infection was defined when two or more infectious pathogens were indentified.

### Statistical analyses

2 × 2 contingency tables were derived to determine sensitivity, specificity, positive predictive value (PPV) and negative predictive value (NPV). All statistics have reported as absolute values with their 95% confidence interval (95% CI). Comparative analysis was conducted by the McNemar test. Data analyses were performed using SPSS 22.0 software. *P* values < 0.05 were considered significant and all tests were 2-tailed.

## Results

### Patient characteristics and mNGS results

A total of 55 patients were enrolled, including 31 males and 24 females, with an average age of 45 years (11–74 years). Among them, 33 (33/55 = 60.0%) patients had underlying diseases, including 9 cases of acute lymphoblastic leukemia (ALL), 9 cases of acute myeloid leukemia (AML), 5 cases of lymphoma, 3 cases of myelodysplastic syndrome (MDS), 3 cases of autoimmune anemia, 2 cases of aplastic anemia, 1 case of chronic myeloid leukemia and 1 case of vasculitis. A total of 36 (36/55 = 65.5%) patients were clinically diagnosed with mixed pulmonary infections, and 19 (19/55 = 34.5%) patients were diagnosed with non-mixed infections (single infections or infections with unknown pathogens) [see Additional file 2: Table S1].

Pulmonary biopsy and bronchoalveolar lavage fluid were collected for mNGS. The report provided specific sequencing reads of all microorganisms with valid data that can be detected in specimen. *Propionibacterium acnes*, *Micrococcus luteus*, *Malassezia globosa, Lactococcus lactis,* and *Saccharomyces* were not interpreted as pathogens, as they were known as normal flora of the skin or respiratory tract.

### Comparison of mNGS and conventional test in the diagnosis of mixed pulmonary infection

#### Comparison of diagnostic performance for differentiating mixed infection from non-mixed infection

In 55 patients with pulmonary infection, the comparison of mNGS and conventional test is presented in Table 1**.** Mixed pulmonary infection was defined when two or more infectious pathogens were identified. The sensitivity and specificity of diagnosing mixed pulmonary infection by mNGS were 97.2% (95% CI: 83.8–99.9%) and 63.2% (95% CI: 38.6–82.8%) respectively, with NPV and PPV being 92.3% (95% CI: 62.1–99.6%) and 83.3% (95% CI: 68.0–92.5%). The sensitivity and specificity of diagnosing mixed pulmonary infection by conventional diagnostic testing were 13.9% (95% CI: 5.2–31.0%) and 94.7% (95% CI: 71.9–99.7%) respectively, with NPV and PPV being 36.7% (95% CI: 23.8–51.7%) and 83.3% (95% CI: 36.5–99.1%).

**Table 1:** Performance of metagenomic next-generation sequencing (mNGS) and conventional testing in diagnosis of mixed pulmonary infection

Diagnostic testing	Sensitivity % (95% CI)	Specifificity % (95% CI)	PPV % (95% CI)	NPV % (95% CI)
Conventional laboratory-based diagnostic testing	13.9 (5.2–31.0)	94. (71.9–99.7)	83.3 (36.5–99.1)	36.7 (23.8–51.7)
mNGS	97.2 (83.8–99.9)	63.2 (38.6–82.8)	83.3 (68.0–92.5)	92.3 (62.1–99.6)

Abbreviations: *PPV* positive predictive value, *NPV* negative predictive value, *CI* confidence interval

#### Concordance between mNGS and conventional test

**Fig. 1:**
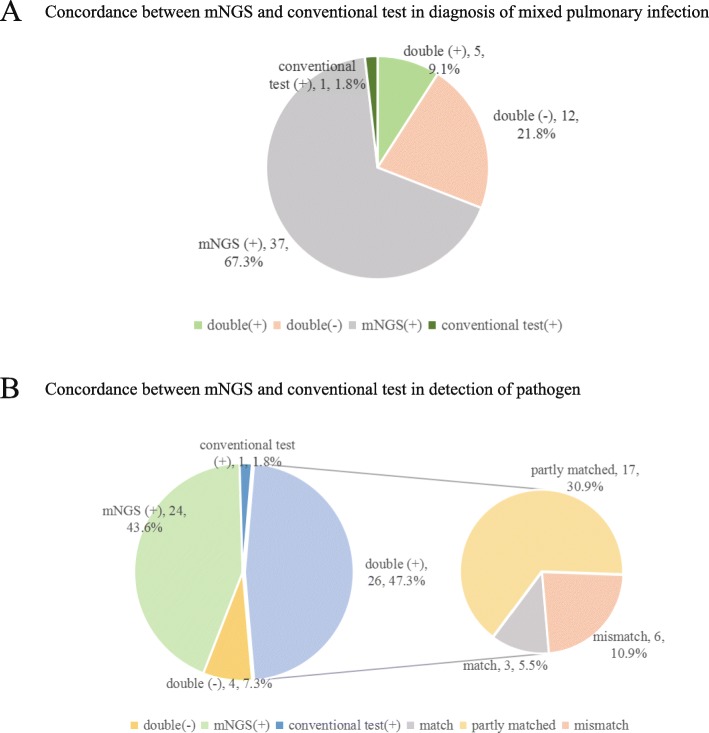
Concordance between metagenomic next-generation sequencing (mNGS) and conventional test. a Pie chart demonstrating the positivity distribution for diagnosis of mixed pulmonary infection by mNGS and conventional test in 55 cases. b Pie chart demonstrating the positivity distribution for detection of pathogen by mNGS and conventional test in 55 cases. For the double-positive subset, a high proportion of partial matching (at least 1 pathogen identified in the test was confirmed by the other) (17/55,30.9%) was seen, with 3 (5.5%) complete matches and 6 (10.9%) conflicts between mNGS and conventional test. mNGS: metagenomic next-generation sequencing

In our results, mNGS and conventional test were both positive for mixed infection diagnoses in 5 (5/55 = 9.1%) cases. A total of 37 (37/55 = 67.3%) cases were positive for mixed infection by mNGS only, 7 of them were false positives. There was 1 (1/55 = 1.8%) case negative for mixed infection by mNGS only, and this case was false negative. mNGS and conventional diagnostic testing were both negative for diagnosing mixed infection in 12 (12/55 = 21.8%) cases, one of them were false negative (Fig. 1 a).

mNGS and conventional test were both positive for pathogens detection (single infection and coinfection) in 26 (26/55 = 47.3%) cases and were both negative in 4 (4/55 = 7.3%) cases. A total of 24 (24/55 = 43.6%) cases were positive for pathogens detection by mNGS only and 1 (1/55 = 1.8%) case was positive by conventional diagnostic testing only. Within the 26 double-positive cases, results of mNGS and conventional tests were completely matched in 3 cases and were totally mismatched in 6 cases. Among 3 completely matched cases, only one was mixed infection, the others were single infections. The remaining 17 cases were found to be partially matched, where at least one detected pathogen was overlapped between mNGS and conventional tests (Fig. 1 b).

### Comparison of mNGS and conventional test in the pathogens detection

#### The Eficiency of mNGS in negative cases identified by conventional test

Of 28 cases (Table 2) [see Additional file 2: Table S1] which had negative results by conventional test, mNGS analysis produced negative results in 4 cases, produced monomicrobial detection in 5 cases and polymicrobial detection in 19 cases. Among 24 patients positive for pathogens, mNGS identified 17 species of pathogens. The most frequent detected pathogen was *Human cytomegalovirus* (12 cases), followed by *Pneumocystis jirovecii* (5 cases), *Ralstonia insidiosa* (5 cases), *Acinetobacter baumannii* (5 cases) and *Pseudomonas aeruginosa* (4 cases). Fungi were reported positive from 13 patients. There were 5 cases positive for *Pneumocystis jirovecii* by mNGS. *Human cytomegalovirus* and *Pneumocystis jirovecii* were detected in NO.1 and NO.4. *Human cytomegalovirus, Pneumocystis jirovecii* and *Rhizopus microsporus* were detected in NO.13. *Pneumocystis jirovecii* and *Torque teno virus* were detected in NO.36. NO.46 was positive for *Pneumocystis jirovecii*. There were another 2 specimens contained *Aspergillus fumigatus* in combination with other pathogens. NO.11 was identified as mixed fungal infection (*Aspergillus niger* and *Candida albicans*) by mNGS, in combination with *Acinetobacter baumannii*. Other specimens contained *Rhizopus delemar*, *Aspergillus oryzae*, *Cryptococcus neoformans* and *Rhizopus oryzae* [see Additional file 2: Table S1]. In conclusion, for negative cases identified by conventional test, mNGS raised the efficiency of mixed pulmonary infection diagnosis.

**Table 2:** Results obtained in the analysis of respiratory specimens of patients

	No. Patients (%)
Positive for mixed infection by mNGS	42 (76.4)
Positive for single infection by mNGS	8 (14.5)
Negative for pathogen by mNGS	5 (9.1)
Positive for mixed infection by conventional test	6 (10.9)
Positive for single infection by conventional test	21 (38.2)
Negative for pathogen by conventional test	28 (50.9)

#### The efficiency of mNGS in positive cases for single infection identified by conventional test

Of 21 (21/55 = 38.2%) cases which were identified as single infection by conventional test, mNGS results were consistent with conventional tests in 2 cases. The pathogen detected were *Pseudomonas aeruginosa* and *Acinetobacter baumannii* in NO.51 and NO.53, respectively. Another 13 results were partially matched, among which 6 cases were positive for *Pneumocystis jirovecii*, *Staphylococcus epidermidis*, *Acinetobacter baumannii*, *Pseudomonas aeruginosa* and *Klebsiella pneumoniae* by culturing. However, mNGS detected more pathogens. Specimens from Patient NO.22 and NO.28 had positive GM test results. Besides *Aspergillus*, mNGS also detected *Haemophilus parainfluenzae* and *Human cytomegalovirus* in NO.22 and NO.28, respectively. The cryptococcal capsular polysaccharide antigen test had positive result in NO.20, and mNGS results reported mixed infection of *Cryptococcus neoformans* and *Pneumocystis jirovecii*. Among 3 cases which were positive for *Mycobacterium tuberculosis* by Xpert MTB, 2 cases were positive for *Mycobacterium tuberculosis* and *Human cytomegalovirus* by mNGS, and 1 case was positive for *Mycobacterium tuberculosis* and *Torque teno virus*. The histopathological examination of 1 case detected mold hyphae, with evidence including alveolar septal fibrous tissue hyperplasia, inflammatory exudate necrosis, silk-like structure and positive PAS results. Results of mNGS were positive for *Pneumocystis jirovecii* and *Aspergillus oryzae* (Table 2). In summary, mNGS complemented the diagnosis of mixed pulmonary infection.

Among the other 6 patients, results of conventional tests were paradoxical with that of mNGS. In Patient NO.18, *Cryptococcus neoformans* identified by cryptococcal capsular polysaccharide antigen test was not detected by mNGS, whereas mNGS reported *Klebsiella pneumoniae, Pseudomonas aeruginosa, Haemophilus parainflfluenzae* and *Aspergillus fumigatus*. In addition, histopathology and culturing results were negative. In Patient NO.23, fungus identified by culture was not detected by mNGS, whereas mNGS reported *Klebsiella pneumoniae, Human cytomegalovirus* and *Rhizomucor pusillus*. *Rhizomucor pusillus* is a thermophilic fungus that lives in hot environment and can infect humans and animals. It can cause necrosis of infected tissues and invade nervous system. It is commonly found in lungs of immunocompromised patients, so it was also interpreted as infectious pathogen. In Patient NO.27, *Staphylococcus epidermidis* identified by culturing was not detected by mNGS, whereas mNGS reported *Human cytomegalovirus* and *Pneumocystis jirovecii*. In Patient NO.29, *Aspergillus* identified by GM test was not detected by mNGS, whereas mNGS reported *Haemophilus parainfluenzae* and *Pseudomonas aeruginosa*. In Patient NO.52, *Aspergillus* identified by GM test was not detected by mNGS, whereas mNGS reported *Acinetobacter baumannii* and *Cryptococcus neoformans*. In Patient NO.54, the culture result was *Flavobacterium indologenes*, whereas mNGS reprted *Cryptococcus neoformans* only [see Additional file 3: Table S1].

#### The efficiency of mNGS in positive cases for mixed pulmonary infection identified by conventional test

Of 6 (6/55 = 10.9%) cases which were identified as mixed infection by conventional test, mNGS results were consistent with conventional tests in 1 case. In 4 out of the 6 cases, results of mNGS and conventional tests were partially matched. Patient NO.30 had culturing positive results for *Acinetobacter baumannii* and *Pneumocystis jirovecii*. Besides, human cytomegalovirus nucleic acid test was positive. In addition to above 3 pathogens, mNGS also detected *Aspergillus fumigatus*. The possible reason for the absence of *Aspergillus fumigatus* in culturing was the limited incubation duration. Specimen from Patient NO.32 was positive for *Pseudomonas aeruginosa* by culturing and positive for *Aspergillus* by GM test, whereas mNGS reported *Aspergillus fumigatus, Pseudomonas aeruginosa* and *Streptococcus pneumoniae*. In Patient NO.34, *Human cytomegalovirus* identified by mNGS was not detected by conventional test. *Aspergillus* identified by GM test was not detected by mNGS. In Patient NO.33, *Pneumocystis jirovecii* was positive by culturing. GM test and human cytomegalovirus nucleic acid test were positive. mNGS identified *Pneumocystis jirovecii* and *human cytomegalovirus*, but not *Aspergillus*. Patient NO.55 was positive for *Acinetobacter baumannii* by culturing. But mNGS result of NO.55 was negative (Table 2). In conclusion, even in the specimen where conventional tests identified mixed pulmonary infection, mNGS still played an important role, because it has the ability to identify both common and rare pathogens without any prior hypothesis.

### Pathogens detected by mNGS

In 55 specimen, 5 species of pathogens (*Mycobacterium abscessus, Rhizopus, Haemophilus parainflfluenzae, Rhizomucor pusillus* and *Streptococcus pneumoniae)* were identified by mNGS, but not by conventional tests; however, *Flavobacterium indologenes* was only detected by the conventional test. Among 55 specimens, the most frequently detected pathogen by mNGS was *Human cytomegalovirus*, followed by *Pneumocystis jirovecii, Pseudomonas aeruginosa, Acinetobacter baumannii, Klebsiella pneumoniae* and *Aspergillus fumigatus*. mNGS reported 19 (55.88%) mixed infections containing *Human cytomegalovirus*. *Human cytomegalovirus* and *Pneumocystis jirovecii* were the most commonly detected co-pathogens in the group of polymicrobial pulmonary infection which were detected in 7 cases. In addition, *Human cytomegalovirus* often co-occurred with *Pseudomonas aeruginosa* (4 cases), *Aspergillus fumigatus* (3 cases), *Klebsiella pneumoniae* (3 cases) and *Acinetobacter baumannii* (3 cases). *Pneumocystis jirovecii* was second in frequency of detection, which was reported in 13 (23.6%) cases. *Pseudomonas aeruginosa* often coexisted with *Klebsiella pneumoniae* and this combination was detected in 5 cases which were the second most common co-pathogens in mixed pulmonary infection (Table 3).

**Table 3:** Human cytomegalovirus, *Pneumocystis jirovecii*, Pseudomona aeruginosa, Klebsiella, pneumoniae, Acinetobacter baumannii and Aspergillus fumigatus among 55 patients.

Pathogen	No. (%)	No. Occurrences with					
		Human cytomegalovirus	*Pneumocystis jirovecii*	*Pseudomonas aeruginosa*	*Klebsiella pneumoniae*	*Acinetobacter baumannii*	*Aspergillus fumigatus*
Human cytomegalovirus	22 (40.0)	–	7	4	3	3	3
*Pneumocystis jirovecii*	13 (23.6)	7	–	1	0	1	1
*Pseudomonas aeruginosa*	11 (20.0)	4	1	–	5	2	1
*Klebsiella pneumoniae*	8 (14.5)	3	0	5	–	1	2
*Acinetobacter baumannii*	8 (14.5)	3	1	2	1	–	2
*Aspergillus fumigatus*	7 (12 7)	3	1	1	2	2	–

## Discussion

mNGS offers the possibility of fast pathogen identification without a prior hypothesis of the target. Theoretically, given sufficiently long sequencing lengths, multiple hits to the microbial genome, and a well-annotated reference database, nearly all microorganisms can be uniquely identified [11]. This retrospective study for the first time reported the sensitivity and specificity of mNGS in the diagnosis of mixed pulmonary infection.

Compared to conventional tests, the sensitivity of mNGS was significantly higher (97.2% vs 13.9% of conventional tests; *P <* 0.01), while the specificity of mNGS was lower (63.2% vs 94.7% of conventional tests; *P* = 0.07). For infectious disease diagnosis, Qing Miao et al. reported mNGS increased the sensitivity rate by approximately 15% in comparison with that of culturing (50.7% vs 35.2%; *P <* 0.01), while the specificity rate of mNGS was comparable with that of culture (89.1% vs 85.7% vs; *P =* 0.39) which is inconsistent with our data [6] .This may be due to the fact that false positive rate of mNGS was high in our results, which was 16.7% (95% CI: 7.5–32.0%).

According to our data, mNGS had a broader spectrum for pathogen detection than conventional tests. Most patients (60.0%) enrolled in this study were immunocompromised because of hematological malignancies, and the efficacy of routine culturing (i.e., growth in media) in pathogen detection was hampered by early administration of broad spectrum or prophylactic antimicrobial drugs. The presence of fastidious or slow growing pathogen also limited the sensitivity of culturing-based methods [5]. Application of mNGS improved the diagnosis sensitivity of pulmonary fungal infections. mNGS identified fungi in 31 (56.4%) out of 55 cases, of which only 10 (18.2%) cases were positive for the same fungi by conventional tests. Qing Miao et al. systematically compared detection by mNGS and culturing in a pairwise manner and found that mNGS had superior feasibility in detecting fungi (OR, 4.0 [95% CI, 1.6–10.3]; P<0.01) [6]. In our results, *Rhizopus* identified in 3 cases by mNGS was not detected by any conventional tests. Henan Li et al. reported that tissues were usually homogenized in a glass grinder and used for smear and culture in the clinical microbiology laboratory, and this grinding procedure may affect the isolation of *Zygomycetes* (such as *Rhizopus* and *Mucor*). The mNGS analysis doesn’t require this grinding procedure, and identified more *Zygomycetes* than culturing-based method [7]. The number of cases positive for *Aspergillus* identified by conventional tests (9 cases, 16.4%) was less than the number of cases identified by mNGS (14 cases, 25.5%). *Aspergillus* culturing is time-consuming with low positive rate. The time required for smear to check fungi is short, but operators are supposed to have higher abilities to identify fungi among the same genus. The GM test is highly recommended in the diagnosis of *Aspergillus* [12]. However, there are many controversies in the application of GM test, such as: 1) sensitivity and specificity are varied in different diseases; 2) special types of diseases and patient status can lead to false positive results.

The results of this study indicated that mNGS covered more bacteria. The mixed infection of *Pseudomonas aeruginosa* and *Klebsiella pneumoniae* in this study was the second (5 cases, 13.9%) common combination. The positive rate of *Pseudomonas aeruginosa* and *Klebsiella pneumoniae* by mNGS was higher than that by culturing. The positive rate of other bacteria such as *Mycobacterium tuberculosis, Acinetobacter baumannii and Haemophilus parainflfluenzae* by mNGS was also higher than that by culturing. However, Toma et al reported that, compared with sequencing, culturing-based method is able to identify the vast majority (74%) of bacterium-associated pneumonia [13]. The inconsistence between our study and Toma’s might result from the low immune functions of most patients in this study. The use of prophylactic or broad-spectrum antibiotics made bacterial culture even more difficult.

In this study, the underlying diseases of 23 patients were hematological malignancies with low immune functions. Thus, pathogens of mixed infections in these patients might be different from those in the general population. *Human cytomegalovirus* was the most commonly detected pathogen in the study, which occurred in 19 cases of mixed infection. Of these 19 patients, only 2 patients were positive for *Human cytomegalovirus* by conventional tests, and 17 patients were positive by mNGS. We also detected mixed infections of *Human cytomegalovirus* and *Pneumocystis jirovecii* in 7 patients, which was the most common combination of pathogens. Immunocompromised patients are susceptible to infection by these pathogens. *Human cytomegalovirus* is a common β-herpesvirus that infects most of the adult population. It remains predominantly dormant after primary infection, and is relatively innocuous in healthy adults [14]. However, in patients with immune dysfunction or immunosuppression, such as acquired immune deficiency syndrome (AIDS) patients, organ transplantation recipients, and patients in the intensive care unit (ICU) [15], *Human cytomegalovirus* infection may cause serious end-stage diseases, such as leukopenia, hepatitis, nephritis, interstitial pneumonia, gastrointestinal disease and even death [16, 17]. *Pneumocystis jirovecii* was an early indicator of the human immunodeficiency virus (HIV) epidemic and occurred in 70–80% of AIDS patients [18]. There is an increasing population of susceptible non-HIV-infected patients, including those with solid malignancies, solid organ transplantation and the recipients of hematopoietic stem cell transplantation, patients receiving immunosuppressive therapies for autoimmune and inflammatory conditions and those with genetic primary immune deficiency disorders [19]. A national study over the decade 2000–2010 showed an increase in incidence of *Pneumocystis jirovecii* infection, and the largest population associated with *Pneumocystis jirovecii* were those suffering from underlying hematological malignancy [20]. The difficulty in isolating and culturing *Pneumocystis jirovecii* has hindered both diagnosis and research. Several methods using various coculture cell lines were described but failed to attain widespread use [21–24]. The application of mNGS is a promising method for the fast and accurate detection of *Pneumocystis jirovecii.*

Our study had several limitations. In our study, the most common co-pathogens in mixed infections were *human cytomegalovirus* and *Pneumocystis jirovecii*, while the majority of patients were immunocompromised, which may lead to biased conclusions. Moreover, our mNGS tests were delivered to the commercial laboratory rather than an microbiology laboratory in hospital, which might sacrifice sensitivity rate because of reduced viability due to increased turnaround time.

## Conclusion

However, we believe that mNGS can identify pathogens (e.g., MTB, viruses, anaerobic bacteria, and fungi) earlier and more comprehensively in mixed pulmonary infection, providing information for improvement of culturing conditions and making antibiotic regimens. mNGS can be a promising technique for accurate diagnosis and customized treatment of mixed pulmonary infection.

## Supplementary information

**Additional file 1:** **Figure S1.** Diagnostic flow chart for mixed pulmonary infection.

**Additional file 2:** **Table S1.** Patients for metagenomic next-generation sequencing (mNGS) and conventional laboratory-based diagnostic testing.

**Additional file 3: Table S1.** The detail information of cases with mismatched results by next-generation sequencing (mNGS) and conventional testing.

## Data Availability

The datasets analysed during the current study are available from the corresponding author on reasonable request.

## Abbreviations

AIDS: Acquired immune deficiency syndrome
ALL: Acute lymphocytic leukemia
AML: Acute myeloid leukemia
BALF: Bronchoalveolar lavage fluid
CI: Confidence interval
GM: Galactomannan
ICU: Intensive care unit
IPA: Invasive pulmonary aspergillosis.
MDS: Myelodysplastic syndrome
mNGS: Metagenomic next-generation sequencing
MTB: *Mycobacterium tuberculosis*
MTBC: *Mycobacterium tuberculosis* complex.
NGS: Next-generation sequencing
NPV: Negative predictive value
PAS: Periodic acid-Schiff
PPV: Positive predictive value
