# Supplementary figures and images for: Metagenomic next-generation sequencing for mixed pulmonary infection diagnosis
Source: BMC Pulm Med. 2019 Dec 19;19:252. doi: 10.1186/s12890-019-1022-4 (PMC6921575; doi:10.1186/s12890-019-1022-4)

**Additional file 1** Diagnostic flow chart for mixed pulmonary infection

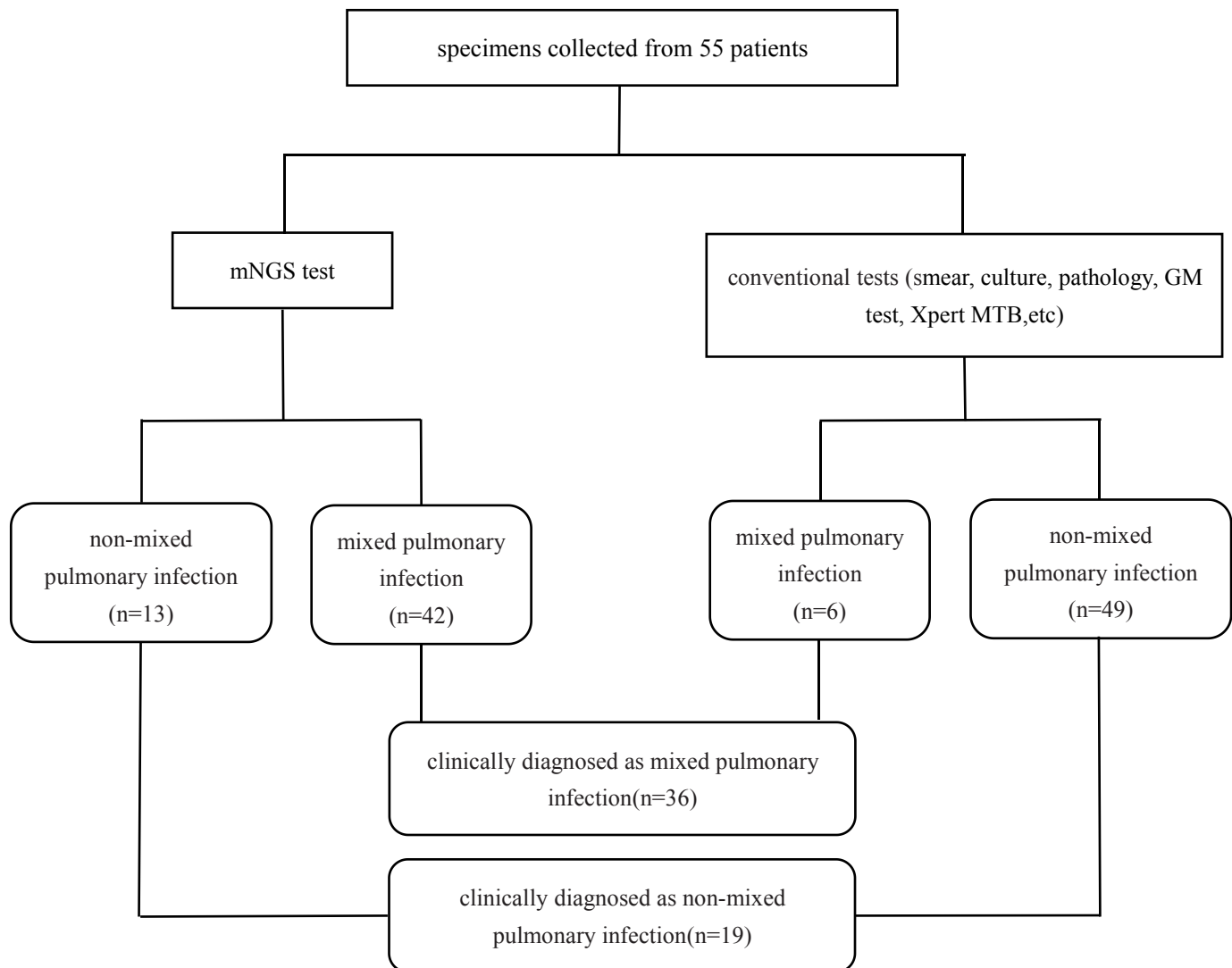

Supplement: Supplementary file 1 — Additional file 1: Figure S1. Diagnostic flow chart for mixed pulmonary infection. [file 12890_2019_1022_MOESM1_ESM.pdf]
