# Supplementary material for: Metagenomic next-generation sequencing for mixed pulmonary infection diagnosis
Source: BMC Pulm Med. 2019 Dec 19;19:252. doi: 10.1186/s12890-019-1022-4 (PMC6921575; doi:10.1186/s12890-019-1022-4)
Supplement: Supplementary file 2 — Additional file 2: Table S1. Patients for metagenomic next-generation sequencing (mNGS) and conventional laboratory-based diagnostic testing. [file 12890_2019_1022_MOESM2_ESM.docx]

**Additional file 2** Patients for metagenomic next-generation sequencing (mNGS) and conventional laboratory-based diagnostic testing.

| Patient ID | Underlying disease | Pulmonary disorders | Smear results | Culture results | Pathology results | GM test | Xpert MTB | mNGS based diagnosis  (number of unique reads) | Other results |
| --- | --- | --- | --- | --- | --- | --- | --- | --- | --- |
| NO.1 | Immunological anemia | pulmonary infection,  Type Ⅰ respiratory failure | Negative | Negative | Alveolar septal fibrous tissue hyperplasia,  inflammatory cell infiltration | Negative | Negative | *Human cytomegalovirus*  (72)*,*  *Pneumocystis jirovecii*(28) | Negative |
| NO.2 | None | pulmonary infection | Negative | Negative | Fibrous tissue hyperplasia,  inflammatory cell infiltration | Negative | Negative | *Pseudomonas aeruginosa*(50)*,*  *Human cytomegalovirus*  (32) | Negative |
| NO.3 | Vasculitis | pulmonary infection | Negative | Negative | Fibrous tissue hyperplasia,  inflammatory exudative necrosis | Negative | Negative | *Mycobacterium abscessus*(2)*,*  *Aspergillus fumigatus*(6) | Negative |
| NO.4 | AML | pulmonary infection | Negative | Negative | Fibrous tissue hyperplasia,  inflammatory cell infiltration | Negative | Negative | *Human cytomegalovirus*  (10)*,*  *Pneumocystis jirovecii*  (50916) | Negative |
| NO.5 | Aplastic anemia | pulmonary infection | Negative | Negative | Interstitial fibrous tissue hyperplasia with inflammatory cell infiltration | Negative | Negative | *Klebsiella pneumoniae*(111)*,*  *Pseudomonas aeruginosa*  (397) | Negative |
| NO.6 | ALL | pulmonary infection | Negative | Negative | Alveolar septal fibrosis, alveolar occlusion, scattered chronic inflammatory cell infiltration | Negative | Negative | *Human cytomegalovirus*  (4)*,*  *Acinetobacter baumannii*(64)*,*  *Aspergillus fumigatus*(12) | Negative |
| NO.7 | None | pulmonary infection | Negative | Negative | Chronic inflammatory cell infiltration, significant proliferation of fibrous tissue leading to alveolar occlusion | Negative | Negative | *Klebsiella pneumoniae*(1)*,*  *Rhizopus oryzae*(1) | Negative |
| NO.8 | MDS | pulmonary infection | Negative | Negative | Alveolar septal fibrous tissue hyperplasia,  Inflammatory cell infiltration | Negative | Negative | *Klebsiella pneumoniae*(3)*,*  *Human cytomegalovirus*  (2474)*,*  *Pseudomonas aeruginosa*(55) | Negative |
| NO.9 | ALL | pulmonary infection | Negative | Negative | Alveolar septum widening, interstitial fibrous tissue hyperplasia with scattered inflammatory cell infiltration | Negative | Negative | *Aspergillus niger*(10)*,*  *Human cytomegalovirus*  (103) | Negative |
| NO.10 | None | pulmonary infection | Negative | Negative | Chronic inflammatory cell infiltration, interstitial fibrous tissue hyperplasia, partial alveolar epithelial hyperplasia | Negative | Negative | *Cryptococcus neoformans* (2)*,*  *Human cytomegalovirus*  (5) | Negative |
| NO.11 | None | pulmonary infection,  Type Ⅰ respiratory failure | Negative | Negative | Interstitial fibrous tissue hyperplasia with inflammatory cell infiltration | Negative | Negative | *Acinetobacter baumannii*(61)*,*  *Aspergillus niger*(3)*,*  *Candida albican*(62) | Negative |
| NO.12 | Severe aplastic anemia | pulmonary infection | Negative | Negative | Alveolar epithelial dysplasia | Negative | Negative | *Human cytomegalovirus*  (850)*,*  *Aspergillus oryzae*(134)*,*  *Pseudomonas aeruginosa*(48)*,*  *Acinetobacter baumannii*(5) | Negative |
| NO.13 | Autoimmune hemolytic anemia | pulmonary infection | Gram-  negative bacilli | Negative | Chronic inflammation of the mucosa, a small amount of neutrophil infiltration, partial alveolar septum widening with fibrous tissue hyperplasia | Negative | Negative | *Pneumocystis jirovecii*(64)*,*  *Human cytomegalovirus*  (23282),  *Rhizopus microsporus*(6) | Negative |
| NO.14 | AML | pulmonary infection | Negative | Negative | Fibrous tissue and alveolar epithelial hyperplasia, scattered chronic inflammatory cell infiltration, macrophage visible in some alveolar spaces | Negative | Negative | *Rhizopus delemar*(3168)*,*  *Haemophilus parainflfluenzae*  (9) | Negative |
| NO.15 | AML | pulmonary infection | Negative | Negative | Alveolar septal fibrous tissue hyperplasia, inflammatory exudate necrosis and silk-like structure, histochemical staining showed PAS positive, hexamine silver negative, considering mold hyphae | Negative | Negative | *Pneumocystis jirovecii*(6)*,*  *Aspergillus oryzae*(48) | Negative |
| NO.16 | ALL | pulmonary infection | Negative | *Pneumocystis jirovecii* | Lymphocyte, plasma cell and neutrophil infiltration | Negative | Negative | *Human cytomegalovirus*  (154)*,*  *Pneumocystis jirovecii*(1058) | Negative |
| NO.17 | Chronic myelogeous leukemia | pulmonary infection | Gram-  positive cocci | *Staphylococcus epidermidis* | Interstitial fibrous tissue hyperplasia with inflammatory cell infiltration | Negative | Negative | *Staphylococcus epidermidis*(23)*,*  *Aspergillus fumigatus*(13) | Negative |
| NO.18 | None | pulmonary infection | Negative | Negative | Interstitial fibrous tissue hyperplasia with inflammatory cell infiltration | Negative | Negative | *Klebsiella pneumoniae*(3)*,*  *Pseudomonas aeruginosa*(85)*,*  *Haemophilus parainflfluenzae*  (17)*,*  *Aspergillus fumigatus*(2) | *Cryptococcus neoformans capsular polysaccharide*（+）  *Cryptococcus neoformans capsular polysaccharide* antigen（+） |
| NO.19 | Lymphoma | pulmonary infection | Negative | *Acinetobacter baumannii* | A little carbon dust in the alveolar space | Negative | Negative | *Pseudomonas aeruginosa*(4)*,*  *Acinetobacter baumannii*(2778)*,*  *Klebsiella pneumoniae*(46) | Negative |
| NO.20 | Autoimmune hemolytic anemia | pulmonary infection | Negative | Negative | Alveolar septal fibrous tissue hyperplasia,  a large amount of chronic inflammatory cell infiltration | Negative | Negative | *Cryptococcus neoformans*(88) ,  *Pneumocystis jirovecii*(122) | *Cryptococcus neoformans capsular polysaccharide* antigen（+） |
| NO.21 | ALL | pulmonary infection | Negative | *Pseudomonas aeruginosa* | Alveolar septum widening, interstitial fibrous tissue hyperplasia with a small amount of inflammatory cell infiltration | Negative | Negative | *Pseudomonas aeruginosa*(132)*,*  *Pneumocystis jirovecii*  (3086) | Negative |
| NO.22 | ALL | pulmonary infection | Negative | Negative | Fibrous tissue hyperplasia, inflammatory cell infiltration | Positive | Negative | *Haemophilus parainflfluenzae*  (9),  *Aspergillus niger*(577) | Negative |
| NO.23 | AML | pulmonary infection | Gram-  positive cocci | *Candida albican* | Alveolar septal fibrous tissue hyperplasia, scattered inflammatory cell infiltration | Negative | Negative | *Klebsiella pneumoniae*(4858)*,*  *Human cytomegalovirus*  (86),  *Rhizomucor pusillus*  (24088),  *Torque teno virus*  (7) | Negative |
| NO.24 | AML | pulmonary infection | Negative | *Klebsiella pneumoniae* | Alveolar septal local fibrous tissue mild hyperplasia | Negative | Negative | *Klebsiella pneumoniae*(20)*,*  *Human cytomegalovirus*  (1052) | Negative |
| NO.25 | ALL | pulmonary infection | Negative | Negative | Inflammatory cell infiltration and interstitial fibrous tissue hyperplasia | Negative | Positive | *Mycobacterium tuberculosis*(6) *,*  *Human cytomegalovirus*  (8) | Negative |
| NO.26 | AML | pulmonary infection | Negative | Negative | Mild chronic inflammation, mild hyperplasia of alveolar septal fibrous tissue | Negative | Positive | *Mycobacterium tuberculosis*(864),  *Human cytomegalovirus*  (60) | Negative |
| NO.27 | Lymphoma | pulmonary infection | Gram  -positive cocci,  Gram-  negative bacilli | *Staphylococcus epidermidis* | Alveolar fibrous tissue hyperplasia | Negative | Negative | *Human cytomegalovirus*  (1626),  *Pneumocystis jirovecii*  (303572) | Negative |
| NO.28 | None | pulmonary infection | Negative | Negative | Small airway mucosa chronic inflammation,  inflammatory exudation with neutrophil infiltration | Positive | Negative | *Human cytomegalovirus*  (218),  *Aspergillus fumigatus*(1) | Negative |
| NO.29 | None | pulmonary infection | Negative | Negative | Inflammatory cell infiltration and interstitial fibrous tissue hyperplasia | Positive | Negative | *Haemophilus parainflfluenzae*  (1066)*,*  *Pseudomonas aeruginosa*  (242) | Negative |
| NO.30 | None | pulmonary infection | Gram-  positive cocci | *Acinetobacter baumannii ,*  *Pneumocystis jirovecii* | Inflammatory cell infiltration and interstitial fibrous tissue hyperplasia | Negative | Negative | *Human cytomegalovirus*  (4)*,*  *Pneumocystis jirovecii*(4522)*,*  *Aspergillus fumigatus*(6)*,*  *Acinetobacter baumannii*  (338) | *Human cytomegalovirus* nucleic acid（+） |
| NO.31 | AML | pulmonary infection | Negative | Negative | Chronic inflammation of small airway mucosa, localized phosphation of the epithelium and interstitial fibrous tissue hyperplasia | Positive | Positive | *Mycobacterium tuberculosis*(78),  *Aspergillus oryzae*(112) | Negative |
| NO.32 | ALL | pulmonary infection | Negative | *Pseudomonas aeruginosa* | Inflammatory exudation and necrosis | Positive | Negative | *Aspergillus fumigatus*(1653)*,*  *Pseudomonas aeruginosa*(103)*,*  *Streptococcus pneumoniae*(3) | Negative |
| NO.33 | None | pulmonary infection | Negative | *Pneumocystis jirovecii* | Inflammatory cell infiltration and interstitial fibrous tissue hyperplasia | Positive | Negative | *Pneumocystis jirovecii*(104054)*,*  *Human cytomegalovirus*  (29) | *Human cytomegalovirus* nucleic acid（+） |
| NO.34 | None | pulmonary infection,  small cell lung cancer | Negative | *Pseudomonas aeruginosa* | Chronic inflammation of small airway mucosa, mild hyperplasia of alveolar septal fibrous tissue, inflammatory cell infiltration | Positive | Negative | *Pseudomonas aeruginosa*  (14978)*,*  *Human cytomegalovirus*  (6) | Negative |
| NO.35 | MDS | pulmonary infection | Negative | Negative | Alveolar septum widening, interstitial fibrous tissue hyperplasia with a small amount of inflammatory cell infiltration | Negative | Negative | Negative | Negative |
| NO.36 | [Diffuse large B cell lymphoma](http://www.youdao.com/w/eng/diffuse_large_b_cell_lymphoma/#keyfrom=dict.phrase.wordgroup) | pulmonary infection | Negative | Negative | Slightly widened alveolar space, interstitial fibrous tissue hyperplasia with a small amount of inflammatory cell infiltration, a little small airway mucosa showing chronic inflammation | Negative | Negative | *Pneumocystis jirovecii*(125)*,*  *Torque teno virus*  (46) | Negative |
| NO.37 | None | pulmonary infection | Negative | Negative | Inflammatory cell infiltration and interstitial fibrous tissue hyperplasia | Negative | Negative | Negative | Negative |
| NO.38 | ALL | pulmonary infection | Negative | Negative | Chronic inflammatory cell infiltration and interstitial fibrous tissue hyperplasia | Negative | Negative | *Human cytomegalovirus*  (2),  *Ralstonia insidiosa*  (205) | Negative |
| NO.39 | AML | pulmonary infection | Negative | Negative | Fibrous tissue hyperplasia | Negative | Negative | *Haemophilus parainflfluenzae*  (2686)*,*  *Candida tropicalis*  (10) | Negative |
| NO.40 | AML | pulmonary infection | Negative | Negative | Fibrous tissue hyperplasia, chronic inflammatory cell infiltration | Negative | Negative | *Ralstonia insidiosa*(36)*,*  *Acinetobacter baumannii*(3) | Negative |
| NO.41 | None | pulmonary infection | Negative | Negative | Fibrous tissue hyperplasia, scattered lymphocyte  infiltration | Negative | Negative | *Ralstonia insidiosa*(37) | Negative |
| NO.42 | MDS | pulmonary infection | Negative | Negative | Slightly widened alveolar space, scattered inflammatory cell infiltration, fibroblast formation in the alveolar cavity | Negative | Negative | *Ralstonia insidiosa*(2028)*,*  *Acinetobacter baumannii*(357) | Negative |
| NO.43 | None | pulmonary infection | Negative | Negative | Interstitial chronic inflammatory cell infiltration, alveolar septal fibrous tissue hyperplasia | Negative | Negative | *Human cytomegalovirus*  (213) | Negative |
| NO.44 | None | pulmonary infection | Negative | Negative | Chronic inflammation, widening of alveolar septum, interstitial fibrous tissue hyperplasia with lymphocytes, plasma cells and eosinophil infiltration | Negative | Negative | *Human cytomegalovirus*  (3) | Negative |
| NO.45 | None | pulmonary infection | Negative | Negative | Chronic inflammation of airway mucosa, widening of the alveolar septum, mild hyperplasia of fibrotic tissue ,  lymphocytic infiltration | Negative | Negative | Negative | Negative |
| NO.46 | None | pulmonary infection | Negative | Negative | Mild hyperplasia of alveolar septal fibrous tissue , lymphocytic infiltration | Negative | Negative | *Pneumocystis jirovecii*  (70) | Negative |
| NO.47 | None | pulmonary infection | Negative | Negative | Chronic inflammation of small airway mucosa, mild fibrosis of lung tissue, multiple focal eosinophil infiltration | Negative | Negative | Negative | Negative |
| NO.48 | None | pulmonary infection | Negative | Negative | Slightly widened alveolar space, interstitial mild fibrous tissue hyperplasia, scattered inflammatory cell infiltration | Negative | Negative | *Aspergillus oryzae*  (14) | Negative |
| No.49 | ALL | pulmonary infection | Negative | Negative | Granulomatous lesions, atypical caseous necrosis and multinucleated giant cells | Negative | Positive | *Human betaherpesvirus 5*  (218),  *Aspergillus fumigatus*  (1) | Negative |
| NO.50 | Lymphoma | pulmonary infection | Negative | *Pneumocystis jirovecii* | Mild hyperplasia of fibrous tissue, a little lymphocyte infiltration | Negative | Negative | *Pneumocystis jirovecii*(86)*,*  *Human cytomegalovirus*  (4) | Negative |
| NO.51 | [Diffuse large B cell lymphoma](http://www.youdao.com/w/eng/diffuse_large_b_cell_lymphoma/#keyfrom=dict.phrase.wordgroup) | pulmonary infection | Negative | *Pseudomonas aeruginosa* | Mild hyperplasia of fibrous tissue in local alveolar septal | Negative | Negative | *Pseudomonas aeruginosa*  (395) | Negative |
| NO.52 | None | pulmonary infection | Negative | Negative | Alveolar septum widening, mild hyperplasia of interstitial fibrous tissue with lymphocytic infiltration | Positive | Negative | *Ralstonia insidiosa*(117)*,*  *Cryptococcus neoformans*(1) | Negative |
| NO.53 | None | pulmonary infection | Negative | *Acinetobacter baumannii* | Mild hyperplasia of alveolar septal fibrous tissue,lymphocyte infiltration | Negative | Negative | *Acinetobacter baumannii*  (128) | Negative |
| NO.54 | None | pulmonary infection | Negative | *Flavobacterium indologenes* | Alveolar septal fibrous tissue hyperplasia,  lymphocyte and neutrophil infiltration | Negative | Negative | *Cryptococcus neoformans*(4) | Negative |
| NO.55 | None | pulmonary infection | Negative | *Acinetobacter baumannii* | Small airway mucosa chronic inflammation,  large amount of inflammatory exudate necrosis | Negative | Negative | Negative | Sputum T-spot（+） |

Abbreviations: *AML* acute myeloid leukemia, *ALL* acute lymphocytic leukemia, *MDS* myelodysplastic syndrome
