# Supplementary material for: Metagenomic next-generation sequencing for mixed pulmonary infection diagnosis
Source: BMC Pulm Med. 2019 Dec 19;19:252. doi: 10.1186/s12890-019-1022-4 (PMC6921575; doi:10.1186/s12890-019-1022-4)
Supplement: Supplementary file 3 — Additional file 3: Table S1. The detail information of cases with mismatched results by next-generation sequencing (mNGS) and conventional testing. [file 12890_2019_1022_MOESM3_ESM.docx]

**Additional file 3** The detail information of cases with mismatched results by next-generation sequencing (mNGS) and conventional testing.

| Patient  ID | Smear results | Culture results | Histopathology results | Other laboratory-based  diagnostic testing results | mNGS | Clinical diagnosis of  pulmonary disorders |
| --- | --- | --- | --- | --- | --- | --- |
| NO.18 | Negative | Negative | Interstitial fibrous tissue hyperplasia with inflammatory cell infiltration | *Cryptococcus neoformans capsular polysaccharide*（+）  *Cryptococcus neoformans capsular polysaccharide* antigen（+） | *Klebsiella pneumoniae,*  *Pseudomonas aeruginosa,*  *Haemophilus parainflfluenzae,*  *Aspergillus fumigatus* | Pulmonary infection |
| NO.23 | Gram-positive cocci | *Candida albican* | Alveolar septal fibrous tissue hyperplasia, scattered inflammatory cell infiltration | Negative | *Klebsiella pneumoniae,*  *Human cytomegalovirus*,  *Rhizomucor pusillus* | Pulmonary infection |
| NO.27 | Gram-positive cocci,  Gram-negative bacilli | *Staphylococcs epidermidis* | Alveolar fibrous tissue hyperplasia | Negative | *Human cytomegalovirus,*  *Pneumocystis jirovecii* | Pulmonary infection |
| NO.29 | Negative | Negative | Inflammatory cell infiltration and interstitial fibrous tissue hyperplasia | GM test（+） | *Haemophilus parainflfluenzae,*  *Pseudomonas aeruginosa* | Pulmonary infection |
| NO.52 | Negative | Negative | Alveolar septum widening, mild hyperplasia of interstitial fibrous tissue with lymphocytic infiltration | GM test（+） | *Ralstonia insidiosa,*  *Cryptococcus neoformans* | Pulmonary infection |
| NO.54 | Negative | *Flavobacterium indologenes* | Alveolar septal fibrous tissue hyperplasia,  lymphocyte and neutrophil infiltration | Negative | *Cryptococcus neoformans* | Pulmonary infection |

GM, galactomannan
